# Pyogenic Spondylitis Caused by Methicillin-Resistant *Staphylococcus aureus* Associated with Tracheostomy followed by Resection of Ossification of the Anterior Longitudinal Ligament

**DOI:** 10.1155/2018/9076509

**Published:** 2018-02-18

**Authors:** Michio Hongo, Naohisa Miyakoshi, Masashi Fujii, Yuji Kasukawa, Yoshinori Ishikawa, Daisuke Kudo, Yoichi Shimada

**Affiliations:** Department of Orthopedic Surgery, Akita University Graduate School of Medicine, 1-1-1 Hondo, Akita 010-8543, Japan

## Abstract

Symptomatic ossification of the anterior longitudinal ligament (OALL) is rare. However, when the osteophyte enlarges and obstructive symptoms occur, the patient may require surgery. We present a case of pyogenic spondylitis caused by methicillin-resistant *Staphylococcus aureus* associated with tracheostomy followed by resection of OALL. A 69-year-old woman with OALL complained of dysphagia and suffocation, which was caused by prominent OALL at C4-5. Tracheostomy was performed, followed by osteophytectomy 6 weeks later. Two months after osteophytectomy, she complained of muscle weakness of the extremities, neck pain, and elevated temperature. Magnetic resonance imaging showed an intensity change at the C4-5 vertebrae and an epidural abscess that was causing cord compression requiring urgent decompression. Cultures identified methicillin-resistant *Staphylococcus aureus*. As osteolytic change and muscle weakness gradually progressed, she underwent anterior and posterior reconstruction with an autograft and instrumentation. Bone union was confirmed at 1 year postoperatively with improvement in neurological status. OALL has potentially the risk of airway obstruction. Therefore, appropriate diagnosis and prompt osteophytectomy are needed in cases of a large prominent ossification that puts the patient at risk of suffocation. However, it is noted that osteophytectomy following urgent tracheostomy carries the possible risk of infection.

## 1. Introduction

Ossification of the anterior longitudinal ligament (OALL) was first described by Forestier et al. [1, 2] as ankylosing hyperostosis of the spine. It is also known as diffuse idiopathic skeletal hyperostosis (DISH) [3]. OALL is usually asymptomatic and so is found incidentally. When the OALL is extraordinarily large, it can compress the pharyngoesophageal and laryngotracheal segments, resulting in several symptoms, including dysphagia, dyspnea, and hoarseness. The incidence of these symptoms, however, is relatively low. Airway obstruction is rare, but when it appears, it can be fatal. Here, we present a case of pyogenic spondylitis caused by Methicillin-resistant *Staphylococcus aureus* associated with tracheostomy followed by resection of OALL. It eventually was complicated with myelopathy caused by ossification of the posterior longitudinal ligament (OPLL).

## 2. Case Report

### 2.1. History and Examination

**Figure 1:**
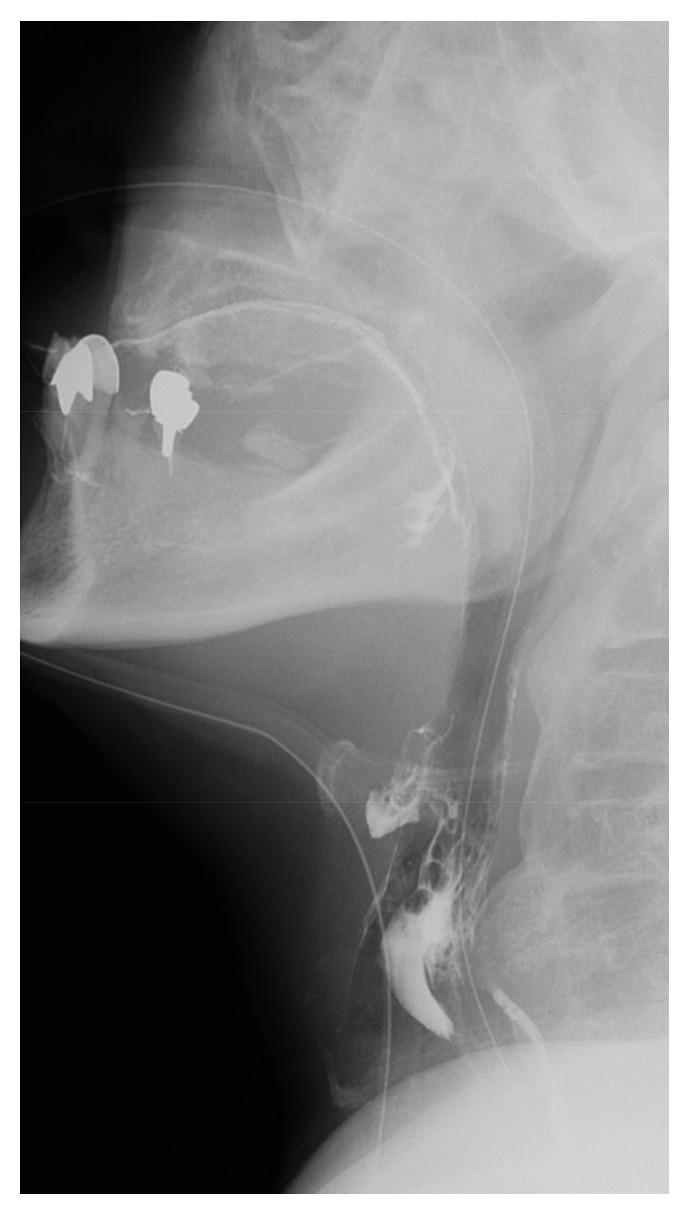
Esophagography showing prominent ossification of OALL compressing the esophagus and trachea.

**Figure 2:**
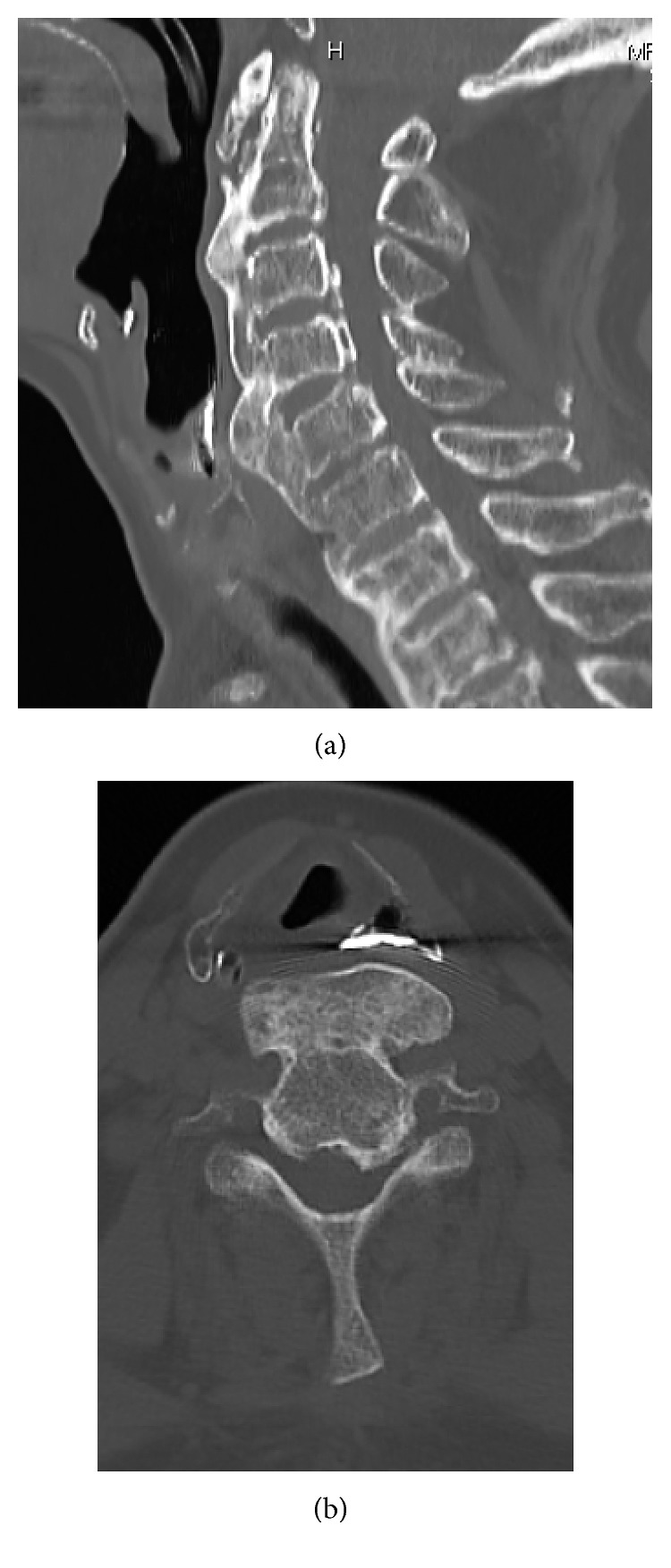
Sagittal (a) and axial (b) image of CT showing extensive OALL at C4-5.

A 69-year-old woman who had had dysphagia for 3 years visited our hospital because of having difficulty drinking. She had a history of type 2 diabetes mellitus, angina, Basedow disease, gallstones, and a left femoral diaphyseal fracture. Firstly, she was suspicious of having neurological dysphagia. However, her neurological examination was within normal limits, according to the evaluation by neurology department. Plain radiographs of the cervical spine showed prominence of OALL at C4-5. Esophagography revealed barium retention at the laryngeal part of the pharynx (Figure 1) and anterior displacement of the esophagus and trachea at C4-5 due to OALL, although swallow motility otherwise appeared normal. Radiographs of the thoracic and lumbar spine were compatible with DISH. Computed tomography (CT) revealed the prominence of the OALL accompanied with displacement of the esophagus at C4-5 (Figures 2(a) and 2(b)).

### 2.2. First Treatment

**Figure 3:**
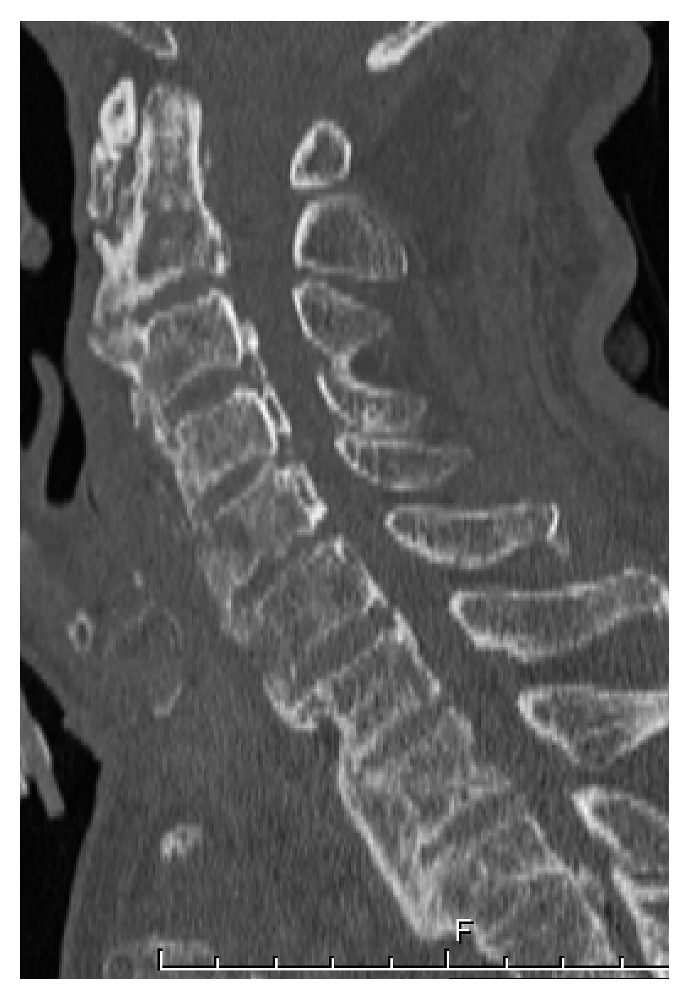
Sagittal CT image after removal of OALL.

We considered surgery to remove the prominent OALL, but the otolaryngologist warned of impending suffocation. Urgent tracheostomy was therefore performed to avoid that situation. Osteophytectomy immediately following tracheostomy offered a potentially high risk of infection because the two skin incisions would be close. Therefore, we waited for the soft tissues to recover. However, her swallowing function did not improve, so, unavoidably, we performed an osteophytectomy using a left anterolateral transcervical approach 6 weeks after the tracheostomy (Figure 3).

### 2.3. Second Treatment

**Figure 4:**
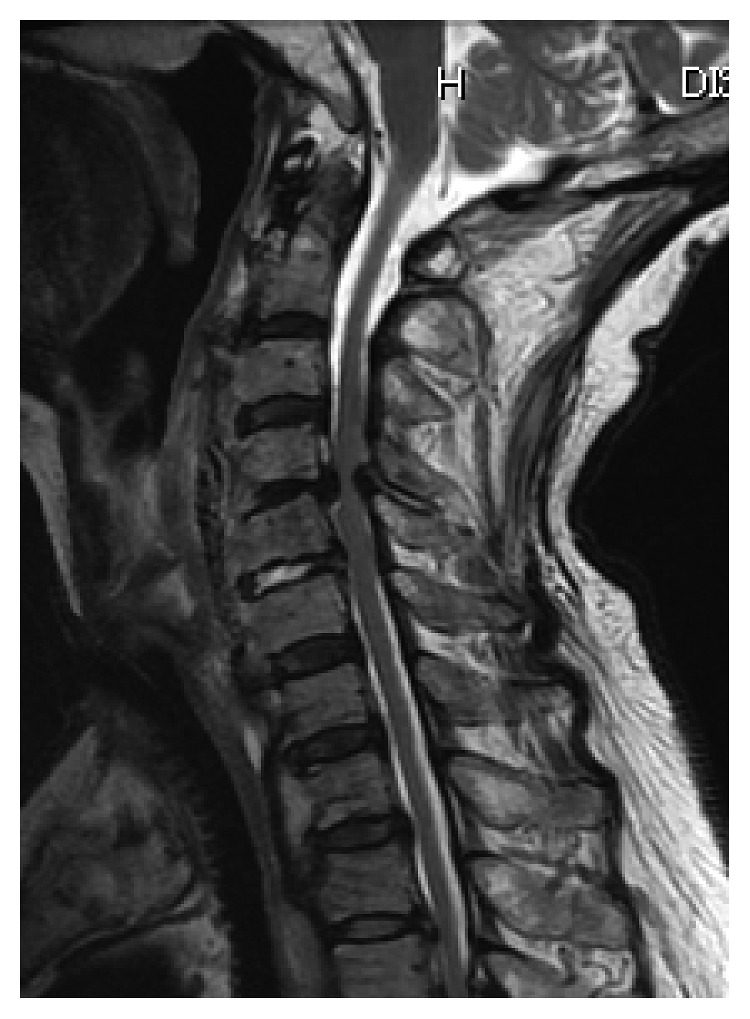
Magnetic resonance imaging after removal of OALL showing spinal canal stenosis due to ossification of the posterior longitudinal ligament at C4-5.

Two months after the OALL resection, she complained of severe neck pain and an elevated temperature. Laboratory studies showed an increased C-reactive protein (CRP) of 7.97 mg/dL and a white blood cell count (WBC) of 11,300 cells/mm^3^. Clinical examination revealed muscle weakness in the upper limbs (shoulder abduction, elbow flexion, and elbow extension of 2/5, 3/5, and 4/5, resp.). Cervical magnetic resonance imaging (MRI) showed spinal stenosis at C4-5 due to ossification of the posterior longitudinal ligament (OPLL) and a signal intensity change of the interverbral disc of C5-6 (Figure 4).

**Figure 5:**
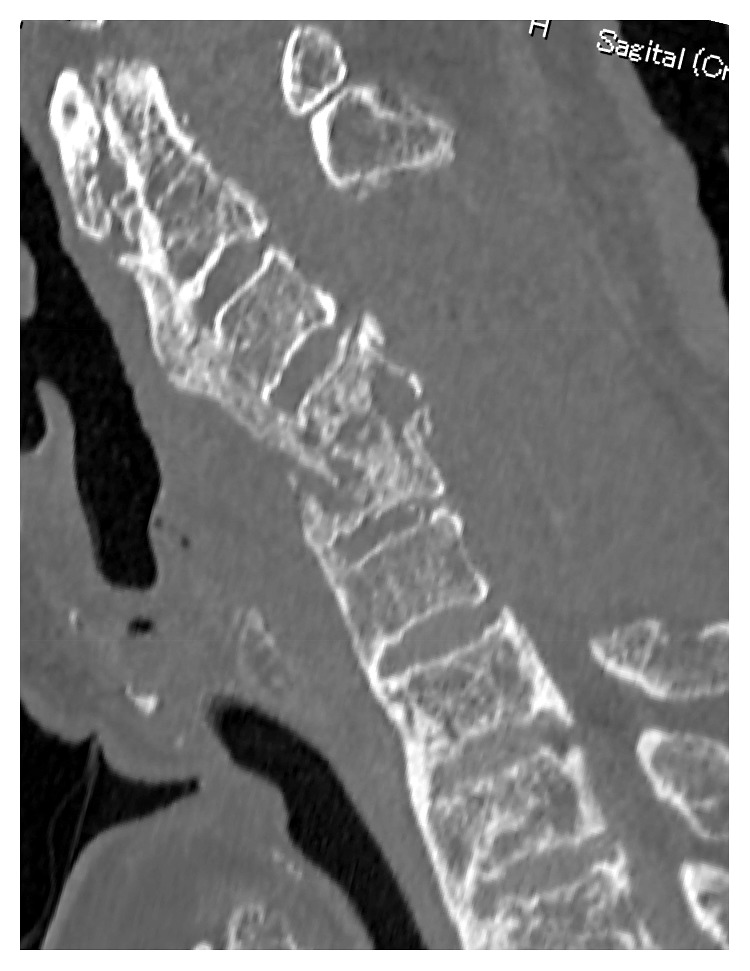
Sagittal CT showing an osteolytic and destructive change at the C4 and 5 vertebra and local kyphosis.

Because her paralysis was progressive, we performed urgent decompression by laminectomy at C3-7. There was no abscess or granuloma caused by infection at the epidural space or posterior column of the cervical spine. After the decompression surgery, she wore a Philadelphia collar, and her muscle strength improved gradually (shoulder abduction, elbow flexion, and elbow extension were 4/5, 4/5, and 4/5, resp.). However, her high body temperature and laboratory evidence of an inflammatory reaction continued. The 6-week follow-up CT findings indicated an osteolytic change at the C4 and 5 vertebra and partial kyphosis at this level (Figure 5). The intensity change on the C4-5 vertebra and the epidural abscess were obvious on MRI with gadolinium contrast.

### 2.4. Third Treatment and Postoperative Course

**Figure 6:**
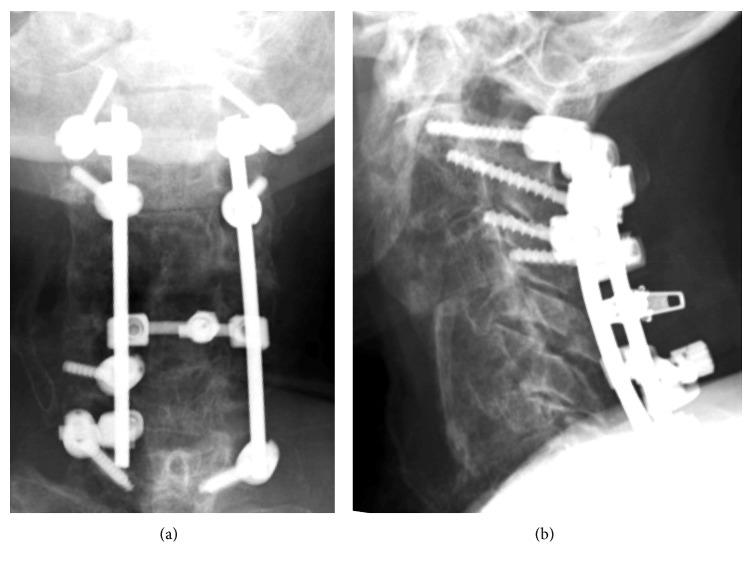
Plain radiography 1 year postoperatively. (a) AP view and (b) lateral view.

We decided to reoperate to remove any infected tissue and reconstruct the cervical spine. She underwent debridement, anterior spinal fusion using iliac bone graft (C3-6), and posterior spinal fusion using lateral mass screws and pedicle screws (C2-7) (Figures 6(a) and 6(b)). She was fitted with a Philadelphia collar for postoperative immobilization.

Intraoperative cultures identified methicillin-resistant *Staphylococcus aureus*. Antibiotic therapy comprised intravenous administration of vancomycin (0.5 g three times daily) and fosfomycin (1 g three times daily), as well as oral administration of rifampicin (150 mg three times daily). At 4 weeks postoperatively, she experienced a recovery of strength and was able to sit up by herself. The laboratory studies normalized, and she was discharged. Spinal stabilization was established one year after the surgery (Figure 6) with obvious bone union confirmed on CT. She showed no deterioration in neurological status.

## 3. Discussion

OALL has been described as a possible cause of dysphagia [4, 5]. The incidence of dysphagia in patients with OALL has been reported to be 17–28% [3, 6, 7]. The various mechanisms of dysphagia are mechanical compression causing esophageal obstruction, pharyngoesophageal irritation, and a local inflammatory response resulting in cricopharyngeal spasm and esophageal denervation [8]. In the present case, no underlying neurological disorders were identified, and the examination of her swallowing function revealed no abnormal movements. Eventually, a prominent OALL was diagnosed as the cause of dysphagia 3 years after the patient's initial complaint and 2 weeks after her presentation to our clinic.

Airway obstruction with or without dysphagia caused by hypertrophic anterior cervical osteophytes is an uncommon pathology, with few reported cases [9–11]. Giger et al. reported a case with progressive dysphagia and acute dyspnea, necessitating emergency tracheotomy [10]. The patient underwent surgical removal of all osteophytes, which led to resolution of the symptoms. Carlson et al. retrospectively investigated nine patients with complaints of dysphagia and who underwent osteophytectomy [11]. Two of the nine patients with dysphagia had simultaneous airway complaints, and one of them required concurrent tracheostomy. In addition to dysphagia and respiratory distress, aspiration pneumonia caused by diffuse cervical hyperostosis was reported [9]. Our patient underwent tracheostomy because of airway obstruction. Careful attention and prompt treatment for failing respiratory function are needed in patients who complain of dysphagia and who have a prominent OALL.

OPLL frequently coexists with OALL, possibly causing spinal cord compression and symptomatic myelopathy. Mizuno et al. reported seven patients with OALL, all of whom complained of dysphagia and underwent removal of the OALL [12]. Five of the patients concurrently had OPLL that required decompression and fusion. Ando et al., although evaluating the surgical outcome of ossification of the thoracic ligamentum flavum (OLF), described the types of OALL that were strongly associated with the severe symptoms and surgical outcomes of OLF [13]. This association was considered to be caused by a mechanical stress shield arising from the OALL. Therefore, under the circumstance of asymptomatic spinal stenosis before the initial surgery in the current case, removal of the prominent ossification and the osteolytic change due to spondylitis increased segmental mobility, leading to worsening of the myelopathy.

Surgical intervention is indicated for patients with respiratory complaints and myelopathy and who failed conservative treatment. The simple removal of OALL through an anterolateral transcervical approach has been shown to alleviate dysphagia [5, 11, 14]. However, several reports recommended that a fusion procedure should accompany removal of OALL to prevent postoperative instability or osteophyte progression and to decompress the spinal cord [15]. Miyamoto et al. reported that surgical resection of osteophytes resulted in a high likelihood of osteophyte recurrence [16].

In the current case, the risk of infection at the surgical site through the anterior approach next to the location of the tracheostomy was still anticipated. Increased risk of postoperative infection in posterior instrumentation for DISH has been reported [17] although tracheostomy did not increase the risk of infection in subsequent anterior cervical surgery in patients with cervical cord injury [18]. Careful preparation of the skin and placement of the second surgical incision lateral to the tracheostomy site should have been prepared. Unavoidable osteophytectomy improved the swallowing function temporarily, but it caused pyogenic spondylitis, which induced instability and resulted in the associated myelopathy. Finally, combined anterior and posterior decompression and fusion with anterior autograft successfully restored the neurological status and eliminated the dysphagia. Solid fusion was achieved once the infected tissues had healed. After reviewing the process of our case, we believe that we could have made an earlier diagnosis of prominent OALL that was likely to cause suffocation. We then would have performed osteophytectomy before tracheostomy.
